# Small cell osteosarcoma versus fusion-driven round cell sarcomas of bone: retrospective clinical, radiological, pathological, and (epi)genetic comparison with clinical implications

**DOI:** 10.1007/s00428-024-03747-2

**Published:** 2024-02-09

**Authors:** Laura S. Hiemcke-Jiwa, Vaiyapuri P. Sumathi, Daniel Baumhoer, Stephanie E. Smetsers, Lianne M. Haveman, Max M. van Noesel, Kirsten van Langevelde, Arjen H. G. Cleven, Michiel A. J. van de Sande, Simone A. J. ter Horst, Lennart A. Kester, Uta Flucke

**Affiliations:** 1 Princess Maxima Center for Pediatric Oncology, Utrecht, The Netherlands; 2 Department of Pathology, University Medical Center Utrecht, Utrecht, The Netherlands; 3 The Royal Orthopaedic Hospital NHS Foundation Trust, Birmingham, UK; 4 Bone Tumor Reference Centre, Institute of Pathology, University Hospital Basel, University of Basel, Basel, Switzerland; 5 Division Imaging & Cancer, University Medical Center Utrecht, Utrecht, The Netherlands; 6 Department of Radiology, University Medical Center Leiden, Leiden, The Netherlands; 7 Department of Pathology, University Medical Center Groningen, Groningen, The Netherlands; 8 Department of Orthopedic Surgery, University Medical Center Leiden, Leiden, The Netherlands; 9 Department of Radiology and Nuclear Medicine, University Medical Center Utrecht, Utrecht, The Netherlands; 10 Department of Pathology, Radboud University Medical Center, Nijmegen, The Netherlands

**Keywords:** Small cell osteosarcoma, Round cell sarcoma, Methylation profiling, Fusion gene analysis

## Abstract

Small cell osteosarcoma (SCOS), a variant of conventional high-grade osteosarcoma (COS), may mimic fusion-driven round cell sarcomas (FDRCS) by overlapping clinico-radiological and histomorphological/immunohistochemical characteristics, hampering accurate diagnosis and consequently proper therapy. We retrospectively analyzed decalcified formalin-fixed paraffin-embedded (FFPE) samples of 18 bone tumors primarily diagnosed as SCOS by methylation profiling, fusion gene analysis, and immunohistochemistry.

In eight cases, the diagnosis of SCOS was maintained, and in 10 cases it was changed into FDRCS, including three Ewing sarcomas (*EWSR1::FLI1* in two cases and no identified fusion gene in the third case), two sarcomas with *BCOR* alterations (*KMT2D::BCOR, CCNB3::BCOR*, respectively), three mesenchymal chondrosarcomas *(HEY1::NCOA2* in two cases and one case with insufficient RNA quality), and two sclerosing epithelioid fibrosarcomas (*FUS::CREBL3* and *EWSR1* rearrangement, respectively).

Histologically, SCOS usually possessed more pleomorphic cells in contrast to the FDRCS showing mainly monomorphic cellular features. However, osteoid was seen in the latter tumors as well, often associated with slight pleomorphism. Also, the immunohistochemical profile (CD99, SATB2, and BCOR) overlapped.

Clinically and radiologically, similarities between SCOS and FDRCS were observed, with by imaging only minimal presence or lack of (mineralized) osteoid in most of the SCOSs.

In conclusion, discrimination of SCOS, epigenetically related to COS, versus FDRCS of bone can be challenging but is important due to different biology and therefore therapeutic strategies. Methylation profiling is a reliable and robust diagnostic test especially on decalcified FFPE material. Subsequent fusion gene analysis and/or use of specific immunohistochemical surrogate markers can be used to substantiate the diagnosis.

## Introduction

High-grade/conventional osteosarcoma (COS) is the most common primary bone malignancy in adolescents and young adults, showing osteogenic differentiation [1, 2]. COS predominantly develops in the lower extremity bones [2, 3] and is often initially identified on radiographs when patients present with pain and swelling, followed by detailed local staging with magnetic resonance imaging using a bone tumor protocol [4].

Diagnostic biopsies, but also (post-treatment) surgical specimens, reveal a broad histomorphological spectrum, including conventional/osteoblastic, chondroblastic, osteoblastoma-like, fibroblastic, telangiectatic, and small cell morphology. They can be pure or mixed and by definition, (mineralized) osteoid is present [2]. Molecularly, COS is characterized by chromothripsis of the genome leading to a complex karyotype [3, 5].

Small cell osteosarcoma (SCOS) is a very rare morphological variant of COS composed of primitive cells with either round or spindle cell morphology [2, 6–8]. In the seminal article, the round cell appearance with resemblance to Ewing sarcoma was emphasized [7]. Later on, two further histological patterns have been observed, namely, large cell lymphoma-like and a small spindle cell variant [6]. Osteoid formation in these tumors is commonly sparse [6, 7]. Furthermore, variable immunohistochemical results may overlap with fusion-driven round cell sarcomas (FDRCS) [9, 10]. Therefore, diagnostic workup of SCOS can cause confusion with the latter, as these sporadically are also reported to contain osteoid [2, 7, 11–14].

Since the treatment protocols for (S)COS are different from FDRCS, accurate diagnosis is paramount for proper treatment strategy [7, 8].

This led us to retrospectively investigate a cohort of 18 cases initially diagnosed as SCOS between 2007 and 2023 by methylation profiling, fusion gene analysis, and immunohistochemistry and correlate epigenetic and genetic findings with clinical, radiological, histopathological, and immunohistochemical characteristics.

## Materials and methods

A total of 18 cases signed out as SCOS between 2007 and 2023 were retrieved from the authors files (four different bone tumor referral centers), including clinical information and formaldehyde-fixed, decalcified, and paraffin-embedded tumor (FFPE). Of all cases, either pretreatment diagnostic biopsies (cases 2–12) or representative tumor from surgical specimens after neo-adjuvant chemotherapy (cases 1 and 13–18) were used for further analyses. 

Radiological images available for five cases were reviewed, and histopathological review of all cases was done to compare SCOS and the different FDRCS when molecular results were received.

All samples were handled according to the ethical guidelines described in “Code for Proper Secondary Use of Human Tissue” in the involved countries in a coded (pseudonymized) manner, as approved by the local institutions IRB.

### Methylation profiling (including copy number variation analysis)

DNA was isolated by NorDiag Arrow using the DiaSorin DNA extraction kit (NL) or GeneRead DNA FFPE Kit (Qiagen) according to the respective manufacturer’s instructions. DNA concentration was measured using the Qubit 2.0 fluorometer. Per sample, we used 500 ng (DK) or 200 ng (NL) of DNA. Bisulfite conversion was performed with EZ DNA Methylation™ Kit (Zymo Research, Irvine, CA, USA). All methylation data were generated using the Illumina® MethylationEPIC (850 k) BeadChip platform as previously described [15]. Classification of the samples was performed by the Heidelberg sarcoma classifier using the most recent version available, either v10.1 or v12.2 [16].

### Whole transcriptome sequencing (mRNA sequencing)

Total RNA was isolated using the AllPrep DNA/RNA/Protein Mini Kit (Qiagen) in keeping with standard protocol on the QiaCube (Qiagen). For cases 1–4, 6, and 7, fresh frozen tissue was used, while in cases 9 and 10, only FFPE was available. RNA-seq libraries were generated with 300 ng RNA using the KAPA RNA HyperPrep Kit with RiboErase (Roche) and subsequently sequenced on a NovaSeq 6000 system (2 × 150 bp) (Illumina). The RNA sequencing data were processed as per GATK 4.0 best practices workflow for variant calling, using a wdl and Cromwell-based workflow (https://gatk.broadinstitute.org/hc/en-us/sections/360007226651-Best-Practices-Workflows). This included performing quality control with Fastqc (version 0.11.5) to calculate the number of sequencing reads and the insert size Picard (version 2.20.1) for RNA metrics output and MarkDuplicates [17]. The raw sequencing reads were aligned using Star (version 2.7.0f) to GRCh38 and gencode version 29 [18].

### Targeted mRNA sequencing

Total RNA was isolated from FFPE sections using the ReliaPrep Total RNA Miniprep system (Promega), and RNA concentrations were measured with the Qubit RNA HS kit (Thermo Fisher Scientific, Waltham, MA). Then, 250 ng RNA input was used for the preparation of cDNA. For preparing open-ended target enriched NGS libraries, anchored multiplex PCR (AMP) technology was applied by using the FusionPlex® kit v1, according to the manufacturer’s instructions (Invitae, San Francisco, CA). A targeted gene panel has been deployed (custom designed), including genes relevant for the differential diagnosis of sarcomas. Library preparation was done according to the manufacturer’s instructions. Pooled libraries were sequenced on a NextSeq 500 (Illumina). Demultiplexing was performed using an in-house bioinformatic workflow, and data were thereafter analyzed using Archer Analysis software (ArcherDX) version 6. All reported analyses were of good quality, defined by the following criteria: > 50% RNA reads and on average ≥ 10 start sites per gene-specific primer targeted to housekeeping genes (SS/GSP2). The reported fusion transcripts were found in the “strong fusion” section of Archer Analysis.

### Reverse-transcription polymerase-chain reaction (RT-PCR)

RT-PCR was performed according to standard procedures as previously described [19].

### Fluorescence in situ hybridization (FISH) analysis

*EWSR1* and *FUS* dual-color FISH were performed on case 17, re-diagnosed as sclerosing epithelioid fibrosarcoma by methylation profiling and without appropriate RNA quality for RNA sequencing. On fresh cut 4 μm sections of FFPE tissue, a break-apart probe for *EWSR1* (Cytocell, Cambridge, UK) and *FUS* (Zytovision, Bremerhaven, Germany) were applied according to the manufacturer’s protocol. At least 50 nuclei per sample were counted.

### Immunohistochemistry

Sections were stained by the use of an automated tissue stainer in the different laboratories. The following antibodies were applied: CD99 (Leica, PCB1, 1:40), BCOR (Zeta corporation, C10, ready to use), SATB2 (Epitomics, AC0268, 1:200), NKX3.1 (Roche, EP356, RTU), SOX9 (Abcam, EPR14335 1:200), NKX2.2 (Pharmingen BP, 74.5, 1:50), and MUC4 (Cell Marque, 8G7, ready to use). Pretreatment was performed according to standard protocols.

## Results

### Molecular results (depicted in Table 1)

**Table 1:** Clinical and molecular characteristics of the study cohort (*n* = 18)

Case	Sex (M/F)	Age (yrs)	Location of primary tumor	Metastases	Follow-up (till July 2023)	Fusion gene analysis	Methylation (calibrated score)
1	F	8	Femur	Femur	DOD (27 months)	NA	High-grade osteosarcoma (0.98)
2	M	49	Tibia	Lung	Unknown	NA	High-grade osteosarcoma (0.97)
3	F	16	Tibia	No	Aw/oD (34 months)	NA	High-grade osteosarcoma (0.99)
4	M	49	Radius	Unknown	Unknown	NA	High-grade osteosarcoma (0.4)
5	M	10	Femur	Lung	DOD (22 months)	NA	High-grade osteosarcoma (0.93)
6	M	63	Scapula	Lung	DOD (21 months)	NA	High-grade osteosarcoma (0.96)
7	M	15	Femur	Lung	DOD (23 months)	NA	High-grade osteosarcoma (0.81)
8	F	40	Femur	Bone, lung	DOD (20 months)	No fusions	High-grade osteosarcoma (0.92)
9	M	17	Fibula	Lymph node	AwD (7 month)	*EWSR1::FLI1 ex11::ex5*	Ewing sarcoma (0.99)
10	F	12	Os sacrum	No	Aw/oD (20 months)	*EWSR1::FLI1 ex8::ex9*	Ewing sarcoma (0.99)
11	F	6	Femur	No	Unknown	No fusions (Archer), no mutations; RT-PCR for Ewing sarcoma also negative	Ewing sarcoma (0.97)
12	M	11	Os ilium	Bone, lung	DOD (37 months)	*KMT2D::BCOR ex43::ex7*	Sarcoma with *BCOR* alteration (0.93)
13	M	12	Left foot	Lung	Aw/oD (25 months)	*BCOR::CCNB3 ex15::ex6*	Sarcoma with *BCOR* alteration (0.99)
14	M	13	Rib	No	Aw/oD (7 months)	*HEY1::NCOA2 ex4::ex14*	Mesenchymal chondrosarcoma (0.99)
15	M	78	Maxilla	Unknown	Aw/oD (8 months)	*HEY1::NCOA2 ex4::ex14*	Mesenchymal chondrosarcoma (0.99)
16	F	40	Vertebrae	No	Unknown	Insufficient RNA quality	Mesenchymal chondrosarcoma (0.627)
17	F	76	Lumbar spine	Lung	Aw/oD (12 months)	Insufficient RNA quality; *EWSR1* break-apart (FISH)	Sclerosing epithelioid fibrosarcoma (0.99)
18	M	60	Unknown	Lung	Unknown	*FUS*::*CREBL3 ex7::ex5*	Sclerosing epithelioid fibrosarcoma (0.99)

Abbreviations: *F*, female; *M*, male; *Aw/oD*, alive without disease; *DOD*, dead of disease; *NA*, not applicable

Methylation profiling was performed successfully in all 18 cases. In eight of them, a diagnosis of high-grade OS was rendered. The other 10 cases were diagnosed as FDRCS including Ewing sarcoma (*n* = 3), sarcoma with *BCOR* alteration (*n* = 2), mesenchymal chondrosarcoma (*n* = 3), and sclerosing epithelioid fibrosarcoma (SEF) (*n* = 2). These diagnoses were confirmed by fusion gene analysis (mRNA sequencing (targeted or whole transcriptome sequencing)), revealing an *EWSR1::FLI1* (*ex11::ex5 and ex8::ex9*) in two out of three Ewing sarcomas, a *KMT2D::BCOR* (*ex43::ex7*) and a *CCNB3::BCOR* (*ex15::ex6*) in the two sarcomas with *BCOR* alteration, a *HEY1::NCOA2* (*ex4::ex14*) in two out of the three mesenchymal chondrosarcomas, and a *FUS::CREBL3* (*ex7::ex5)* in one out of the two SEFs. In one SEF (case 17), RNA quality was insufficient for RNA sequencing. Alternatively performed FISH depicted single red signals (67%) for *EWSR1* interpreted as rearrangement, with *FUS* revealing no break-apart signals. In one of the Ewing sarcomas (case 11), no fusion gene was detected by targeted mRNA sequencing and RT-PCR. There was no material left for *EWSR1* FISH analysis.

The copy number variation (CNV) profiles of all SCOSs were highly variable with multiple gains and losses whereas the FDRCS, including case 11 and case 16 diagnosed as Ewing sarcoma and mesenchymal chondrosarcoma, respectively, without detection of a corresponding fusion gene, had some CNVs but they were neglectable compared to the SCOSs.

### Clinical characteristics (depicted in Table 1)

Of the eight patients whose tumor had a methylation profile of (S)COS, three were female and five were male (cases 1–8). Age ranged from 8 to 63 years (median, 28 years). The tumors were located in the distal femur (*n* = 4), proximal tibia (*n* = 2), radius (*n* = 1), and scapula (*n* = 1). Patients presented with pulmonary (*n* = 5) and/or bone metastases (*n* = 2). Five of them died of metastatic disease after 20–27 months (median 22 months); one was alive without disease after 34 months. Two patients were lost to follow-up.

The three Ewing sarcomas occurred in a 6- and 12-year-old girl (cases 11 and 10) and a 17-year-old boy (case 9). Tumors were located in the femur, sacrum, and fibula. The latter tumor was suspicious for regional lymph node metastases (MRI), and the patient was alive with disease after 7 months. The patient with the sacrum tumor was alive without evidence of disease after 20 months. Of the third patient, no further information was available. The two sarcomas with *BCOR* alteration arose in the ilium and left foot of an 11- and 12-year-old male, respectively (cases 12 and 13). Both patients had metastatic disease with involvement of lung (*n* = 2) and bone (*n* = 1) at diagnosis. One patient died of disease after 37 months, and the second patient was alive without evidence of disease after 25 months. Three tumors turned out to be a mesenchymal chondrosarcoma of a 13- and 78-year-old male (cases 14 and 15) and a 40-year-old female (case 16). Sites were rib, vertebra, and maxillary bone. Two patients were alive without evidence of disease at 7 and 8 months, respectively. No information was available for the patient with the lesion in the spine. The two cases retrospectively diagnosed as SEF were from a 75-year-old female and a 60-year-old male (cases 17 and 18). The lesion of the female patient affected the lumbar spine, and for the latter case, the site of origin was unknown. Both tumors metastasized to the lung. Follow-up, available for the patient with the spine lesion, showed no evidence of disease after 12 months.

### Radiological features (depicted in Table 2)

**Table 2:** Radiological description of cases 9–10, 12–13, and 16

Diagnosis	X-ray	CT	MRI	PET-CT
Ewing sarcoma	Ill-defined, eccentric, osteolytic, irregular periosteal reaction (case 9)	Ill-defined, osteolytic, cortical destruction with soft tissue extension (case 10)	T1: iso-intense (with hyperintense hemorrhagic foci*); T2: heterogeneous hyperintense; after Gd heterogeneous enhancement; strong diffusion restriction; soft tissue extension (cases 9 and 10)	Heterogeneous or high FDG uptake (primary and locoregional lymph node*)
Sarcoma with *BCOR* alteration	Ill-defined, slightly (case 13) and mainly (case 12) sclerotic	NA	T1: iso-intense; T2: hyperintense; osteoid hypo-intense on T1 and T2; after Gd heterogeneous enhancement; high diffusion restriction; permeative, cortical destruction with soft tissue extension (cases 12 and 13)	High FDG uptake (primary and focal bone metastases^)
MCS	NA	Sclerotic (osteoid), osseous destruction inner cortex with hypodense soft tissue component (case 16)	T1: heterogeneous (with hyperintense hemorrhagic collections); T2: heterogeneous, fluid–fluid levels; osteoid hypointense on T1 and T2; moderate to strong diffusion restriction (case 16)	Mild FDG uptake (case 16)

Abbreviations: *Gd*, gadolinium; *FDG*, 18F fluorodeoxyglucose; *MCS*, mesenchymal chondrosarcoma; *NA*, not applicable; *case 9; ^case 12

**Fig. 1:**
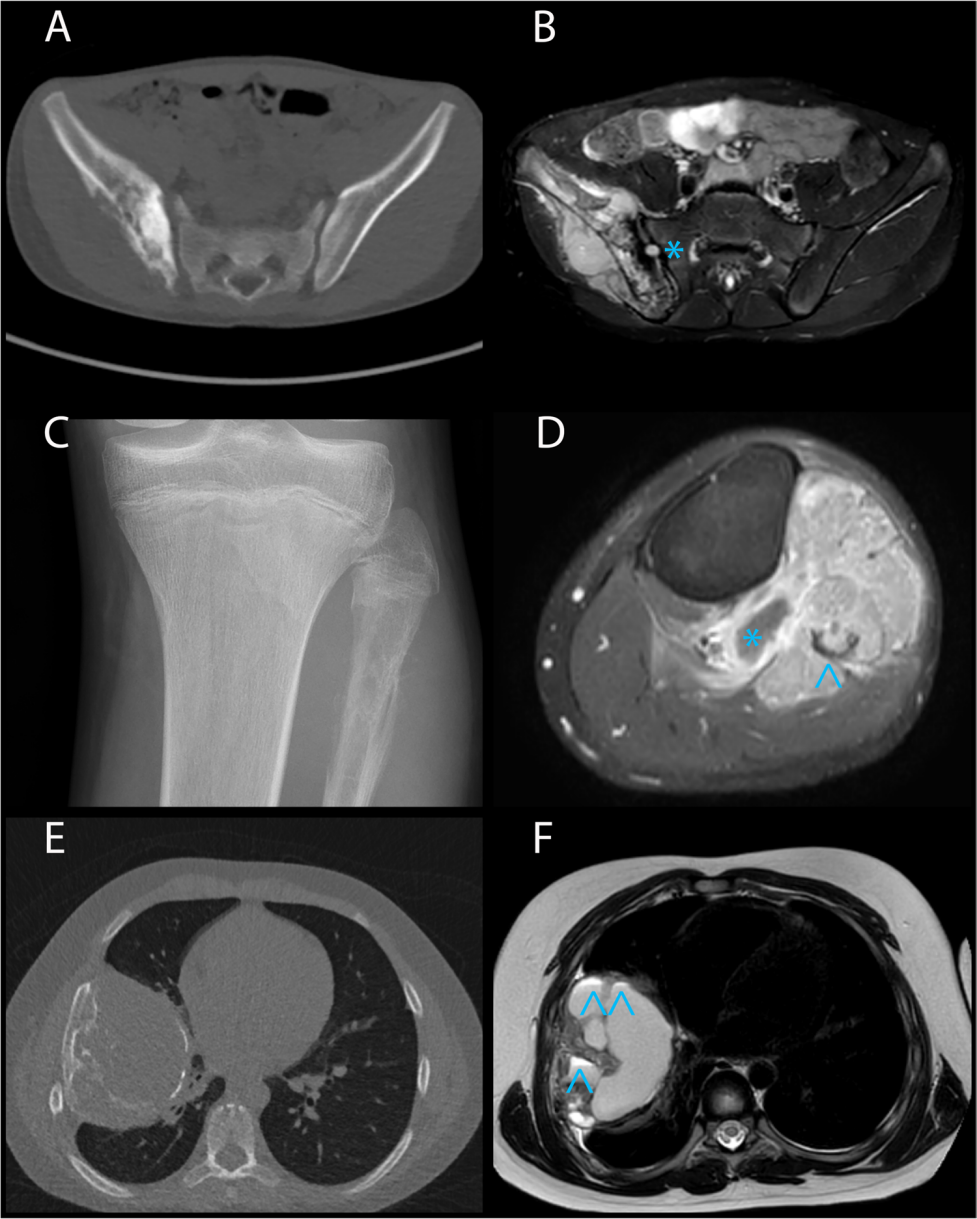
Radiology of FDRCS. **A** Case 12: Non-contrast pelvis CT scan reveals a permeative osseous mass in the right iliac wing with many sclerotic components suggestive for osteoid. **B** Case 12: Transverse T2 SPIR MR sequence shows a T2 heterogeneous mass with a large soft tissue component extending in the right iliac- and gluteal muscles. Note the T2 hypo-intense component lateral to the right SI joint resembling osteoid and the extension into the joint (T2 bright spot in the right SI joint; *). **C** Case 9: On the X-ray of the left knee, an ill-defined osteolytic lesion with irregular periosteal reaction in the proximal metaphysis of the left fibula is observed. **D** Case 9: Transverse T1 SPIR after gadolinium MR sequence depicts a rather homogeneously enhancing mass with large soft tissue component in the anterior- and deep posterior region of the proximal left lower leg. Note the small non-enhancing cystic component (*) and the osseous destruction of the proximal fibula (arrowhead). **E** Case 14: The non-contrast chest CT shows a rounded mass in the right hemithorax with some sclerotic components and osseous deformation and destruction of the inner cortex of the sixth right rib. **F** Case 14: The corresponding transverse T2 MVXD MR sequence depicts the very heterogeneous mass with both homogeneous T2 hyperintense and T2 hypo-intense components. Note the 3 small fluid–fluid levels in the hemorrhagic cystic components (arrowhead)

Radiological characteristics (X-ray, MRI, and CT), available for five FDRCS, are depicted in Table 2 and Fig. 1. Imaging showed an osteolytic lesion in the Ewing sarcomas (cases 9–10) and a sclerotic lesion in the sarcomas with *BCOR* alteration as well as one mesenchymal chondrosarcoma (cases 12, 13, and 16). All Ewing sarcomas and sarcomas with *BCOR* alteration presented with cortical destruction and soft tissue extension, while the mesenchymal chondrosarcoma (case 16), located in the vertebra, invaded the spinal canal. In cases 12, 13, and 16, osteoid was evident. In addition to osteosarcoma, the differential diagnosis included Ewing sarcoma, undifferentiated small round cell sarcoma, lymphoma, giant cell tumor of bone, metastasis of unknown primary, and osteomyelitis.

Using ^18^F-FDG-PET/CT scan, FDG avidity was seen in one Ewing sarcoma and two sarcomas with *BCOR* alteration (cases 10, 12, and 13), while one Ewing sarcoma (case 9) had a heterogeneous signal. Metastases were indicated as shown in Table 1 and 2.

### Histopathology

**Fig. 2:**
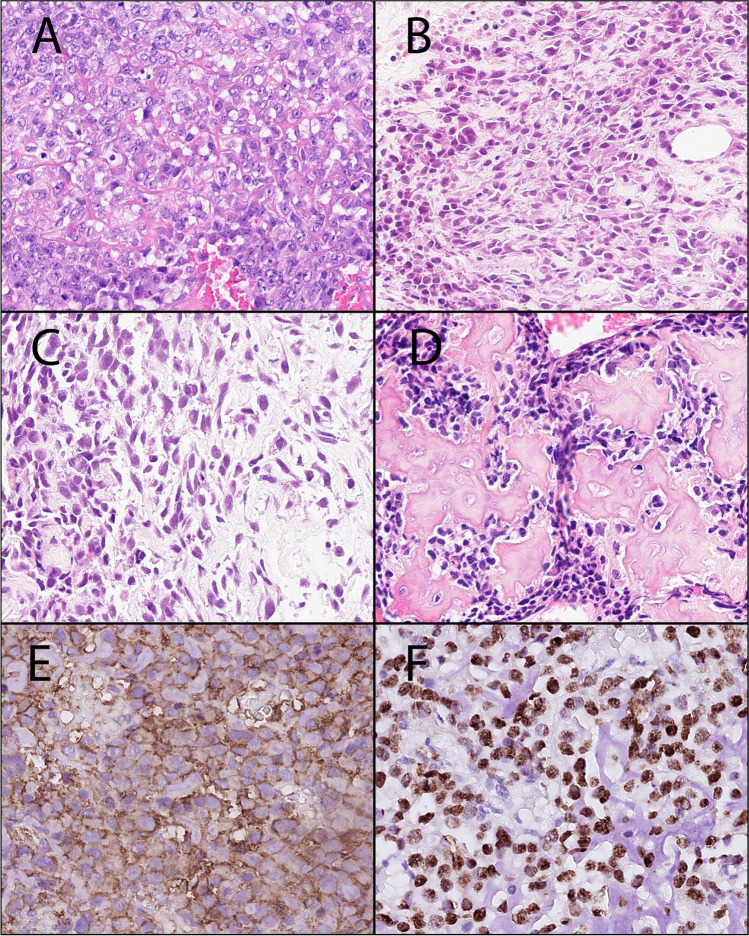
Morphological features (H&E stain) of SCOS cases. **A** Case 1, characterized by pleomorphic tumor cells in an area with deposition of atypical, lace-like osteoid. Magnification 40x. **B–D** Cases 2–4, relatively small but hyperchromatic nuclei and deposition of chondro-osseous matrix. Magnification 40x. **E** Case 1, CD99 immunohistochemistry showing faint and variable membranous staining. Magnification 40x. **F** Case 2, SATB2 immunohistochemistry showing a strong nuclear staining. Magnification 40x

All eight SCOSs displayed sheets of small tumor cells with mainly slight polymorphic, heterochromatic nuclei, and deposition of osteoid, which were only focal, subtle, and lace-like in three cases (Fig. 2). Case 8 had an obvious monomorphic cellular aspect. Necrosis was present in all but one case (case 2). A chondroid matrix was focally detected in case 5.

**Fig. 3:**
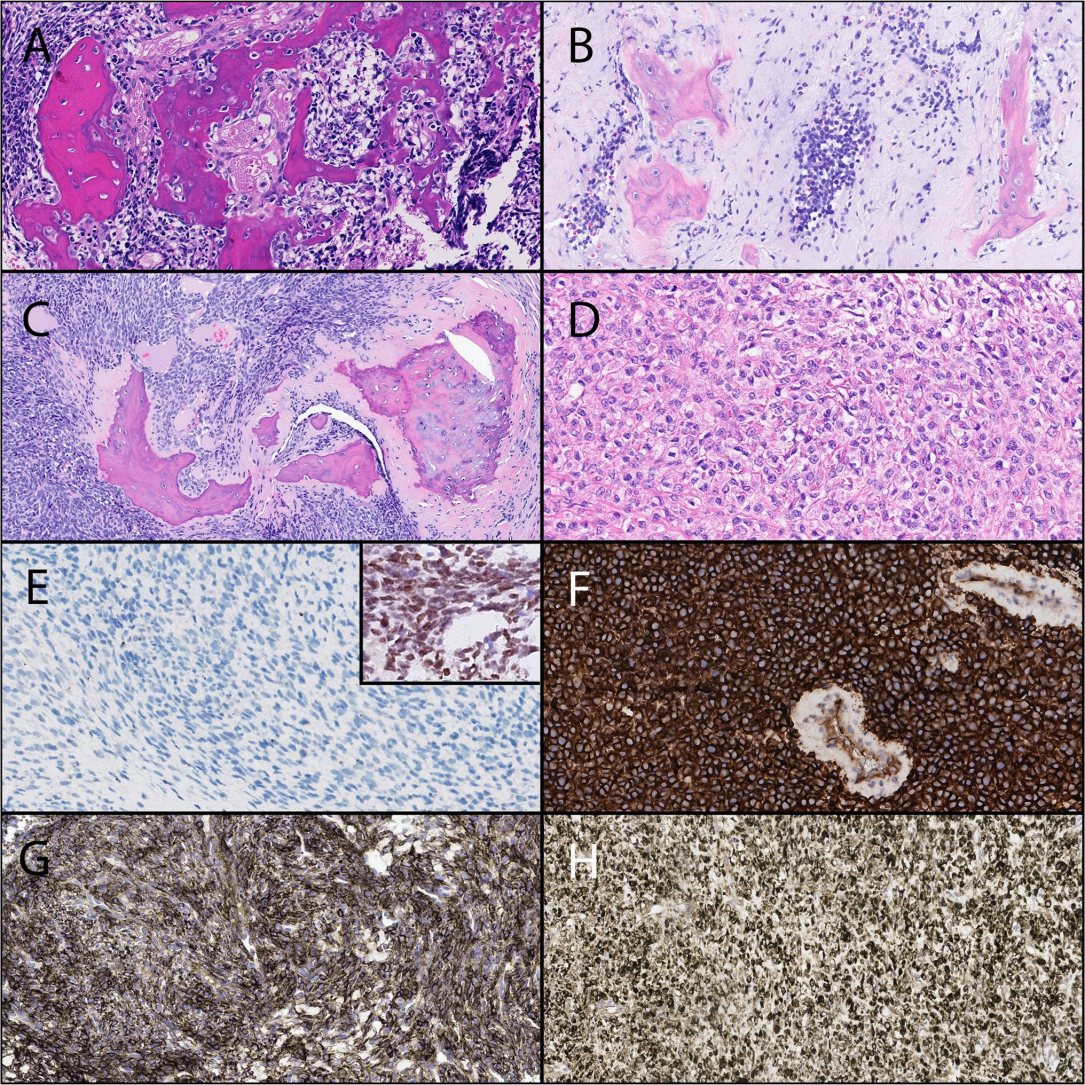
Morphological features (H&E stain) of FDRCS mimicking SCOS. **A** Case 12: Sarcoma with *BCOR* alteration showing a relatively uniform proliferation of round to spindled tumor cells and primitive osteoid deposition. Magnification 32x. **B** Case 9: Ewing sarcoma consisting of monomorphic primitive round to oval tumor cells. Note the (reactive) osseous matrix. Magnification 32x. **C** Case 14. Mesenchymal chondrosarcoma depicting uniform primitive spindle cells and deposition of osteoid with minimal cartilage. Magnification 32x. **D** Case 17: Sclerosing epithelioid fibrosarcoma with epithelioid cells deposited within a delicate collagenous stroma mimicking lace-like osteoid. Magnification 32x. **E** Case 12: BCOR immunohistochemistry in the sarcomas with *BCOR* alteration was negative. Magnification 32x. Inset: SATB2 immunohistochemistry showing nuclear staining in one case tested. **F** Case 9: CD99 immunohistochemistry in a Ewing sarcoma was strong and uniform membranous. Magnification 32x. **G** Case 14: CD99 staining in a mesenchymal chondrosarcoma depicting variable membranous and cytoplasmic positivity. Magnification 32x. **H** Case 17: MUC4 was positive in both sclerosing epithelioid fibrosarcomas. Magnification 32x

The three Ewing sarcomas had a classic undifferentiated small round cell appearance with homogeneous chromatin and small nucleoli [2]. In cases 9 and 10, there was a subtle osteoid (Fig. 3), while in case 11, only small calcifications were observed. In two cases (cases 10 and 11), a prominent fibromyxoid stroma reaction was seen.

The two sarcomas with *BCOR* alteration consisted of small tumor cells with oval to spindled nuclei with an open chromatin. Deposition of atypical osteoid was observed in both cases, and tumor cells in these areas were more polymorphic with hyperchromatic nuclei (Fig. 3). Case 12 showed prominent, dilated vessels. Necrosis was present in both cases.

The three mesenchymal chondrosarcomas comprised monomorphous tumor cells with oval to spindled nuclei with an open chromatin. In all cases, dens collagen deposition was focally seen. In cases 14 and 15, a prominent osteoid matrix with only minor chondroid foci existed (Fig. 3). In these areas, tumor cells had a more polymorphic appearance with heterochromatic nuclei. In case 16, the matrix was chondromyxoid and fibrous without evident calcification. Prominent hemangiopericytoma-like vessels were only seen in case 14. Necrosis was absent in all three cases.

The two SEFs consisted predominantly of sheets of monomorphous small cells with round nuclei possessing an open chromatin, compatible with classic SEF morphology [2]. In both cases, there was a dense collagenous matrix with focal osteoid deposition (Fig. 3). In the latter areas, the tumor cells had a more polymorphic appearance with hyperchromatic nuclei. Necrosis was present in both cases.

### Immunohistochemical results

Two Ewing sarcomas (cases 9 and 10) showed a strong membranous and cytoplasmic CD99 positivity, while in case 11, the staining pattern was heterogeneous. In this case, without an identified fusion gene, a diffuse and strong nuclear expression of NKX2.2 was seen. Both sarcomas with *BCOR* alteration (cases 12 and 13) were negative for BCOR. Case 12 was stained with SATB2 and CD99 (weak). Of the tumors reclassified as mesenchymal chondrosarcomas (cases 14 and 16), CD99 was strongly positive in case 14, while SATB2, BCOR, and MUC4 were negative. Case 16 showed a strong and diffuse nuclear expression of SOX9 and a scattered weak nuclear expression of NKX3.1, while the CD99 reaction was focal and weak. Both SEFs (cases 17 and 18) strongly expressed MUC4.

## Discussion

This study is a reappraisal of 18 cases initially diagnosed as SCOS, with respect to its differential diagnoses of FDRCS. Based upon molecular analyses (methylation profiling and fusion gene analysis), in over 50% of our cases, the initial diagnosis was changed into Ewing sarcoma, sarcoma with *BCOR* alteration, mesenchymal chondrosarcoma, and SEF, emphasizing overlapping clinical, radiological, and pathological features and implicating possible inappropriate treatment for a subset of patients. By CNV analysis, the SCOSs harbored an extensive aberrant genome similar to the morphologically classical COSs, in line with the literature reporting a complex aneuploid karyotype as well as random translocations and mutations in most cases [5, 15]. Round cell sarcomas, on the other hand, usually have a simple karyotype and fusion gene-related translocations leading to chimeric oncoproteins with dysregulated transcription [5, 20]. Affected cells of the latter might be derived from multipotent precursor cells, giving the opportunity to arise in any part of the body [21]. Nevertheless, Ewing sarcomas, sarcomas with *BCOR* alteration, and mesenchymal chondrosarcomas frequently originate in bone with a broad site distribution but a preference for the long bones in Ewing sarcomas and sarcomas with *BCOR* alteration, as is also seen in (S)COS [2, 6, 7, 21–23]. This is reflected by our study showing a broad anatomic distribution of FDRCS in contrast to the SCOSs, which were located mainly in the lower extremity long bones. The metastatic pattern, however, was similar with both groups affecting bones and lung in agreement with the literature [2, 6, 7].

Radiologically, there are similarities between SCOS and FDRCS. Whereas the majority of COS is located in the metaphysis of long bones, depicting mixed osteolytic and sclerotic matrix formation and cortical destruction with a sunburst periosteal reaction, this is not always the case in SCOS. Imaging of SCOS can therefore be difficult to interpret, especially when located atypically, e.g., in the diaphysis, and when showing a mainly lytic appearance without evident mineralization due to more subtle and lace-like bone formation [2, 7]. This overlaps with Ewing sarcoma, sarcoma with *BCOR* alteration, mesenchymal chondrosarcoma, and SEF, also in our cohort [2, 7, 24, 25]. Nevertheless, the presence of (mineralized) osteoid outside the bone is suspicious for COS [6].

As documented in the early descriptions, histomorphologically, these tumors display overlapping features as well [6, 7, 10, 26, 27]. However, SCOS usually shows more variation in nuclear size and commonly a heterochromatic chromatin structure, in contrast to FDRCS most often presenting with monomorphic cells with a more even and open chromatin, with some exceptions [2, 7, 10, 28, 29]. Deposition of (metaplastic) osteoid or a dense collagenous matrix sometimes present in FDRCS can mimic the subtle (lace-like) neoplastic osteoid of SCOS (Fig. 3) [2, 6, 7, 11–14], as seen in our cases. In addition, in the osteoid-presenting areas of our FDRCS, the tumor cells had a more pleiomorphic appearance with hyperchromatic nuclei analogous to SCOS. In agreement with the literature, in some of our SCOSs, subtle morphological transition to COS was apparent, being the morphological clue for assignment as COS [6, 7]. Serial sections of pretreatment biopsies can help to find the diagnostic clues.

Three of our cases turned out to be Ewing sarcomas. Although in one case (case 11) no fusion gene was detected, positive NKX2.2 staining supported the result of methylation profiling. Ewing sarcoma, the prototypical undifferentiated small round cell sarcoma of bone, usually presents with uniform small cells with round nuclei and inconspicuous cytoplasm. Infrequently, the occurrence of larger cells with nuclear variability can be misleading [2, 7, 21]. Also, remodeling of bone intermingled with tumor can lead to misinterpretation as neoplastic osteoid of SCOS, especially in biopsies. SCOS typical lace-like osteoid is rarely present [7, 8, 14]. CD99, showing a distinct membranous staining pattern in Ewing sarcoma, can exhibit diffuse cytoplasmic staining in SCOS [9].

Sarcomas with *BCOR* alteration are composed of round and/or spindle cells with uniform nuclei arranged in sheets when round cells are present and in fascicles and whorls when spindle cells predominate. Pleomorphism is rare and often associated with recurrences. There is variable production of a collagenous and myxoid matrix [28, 29]. Osteoid as seen in our cases is unusual and misleading and is more often reported in metastases [10, 29]. In our cases, tumor cells in areas of osteoid deposition were more pleomorphic complicating the morphological assessment. Additionally, immunohistochemical profiles may overlap with (S)COS with diffuse SATB2 expression and unreliable, sometimes negative BCOR staining also observed in our neoplasms [10].

In mesenchymal chondrosarcoma, the small cell component can be round or spindly and is arranged in sheets or short fascicles. It is intermixed with variable islands of cartilage showing differentiation into mature cartilage with ossification. The cartilage combined with the osteoid can easily be confused with a chondroblastic component of COS or even SCOS when only subtle bone formations are visible as seen in two of our cases [6, 7]. However, the cartilage in mesenchymal chondrosarcoma is well differentiated in appearance in contrast to COS [6, 22]. Also, some variability in cell shape in mesenchymal chondrosarcoma can lead to the misdiagnosis of SCOS. Mesenchymal chondrosarcoma typical hemangiopericytoma-like vessels, one of the diagnostic clues, are variably present and can be absent [30]. SOX9, showing a homogeneous nuclear expression in mesenchymal chondrosarcoma, is negative in SCOS, which can be helpful for discrimination, as demonstrated in one of our cases [22]. Recently, NKX3.1 has been described as a useful marker for mesenchymal chondrosarcomas [31, 32], but it seems that this marker may be expressed in COS [31].

SEF originates mainly in soft tissue and rarely in bone [2, 24–26]. Classically, lesions consist of bland monomorphic epithelioid cells arranged in cords, nests, and sheets within a dense collagenous, commonly hyalinized matrix which can be easily misinterpreted as primitive osteoid, especially when cells are more pleomorphic [24, 25]. SEF is closely related to low-grade fibromyxoid sarcoma (LGFMS) with hybrid cases showing areas of bland-looking spindle cells in addition to areas of classical SEF morphology. Such hybrid cases are documented in bone as well [25]. Chondro-osseous differentiation is exceptionally observed, corresponding to both our cases exhibiting subtle mineralization of the collagenous matrix [33]. The immunohistochemical key marker MUC4, positive in LGFMS and most of the SEFs, as seen in our cases, is usually negative in SCOS [2]. In contrast, positivity for SATB2, reported in some SEFs, can lead to confusion with SCOS [25]. The detection of the canonical fusion genes, *EWSR1/FUS::CREBL1/L2*, naturally rules out SCOS [24–26, 33]. However, prior to the general acceptance of the LGFMS/SEF spectrum, one case of a “SCOS” with a *EWSR1::CREBl1/L2* was reported, underpinning the morphological similarities of SCOS and SEF [34].

Other differential diagnoses of SCOS are mentioned in Table 3, including synovial sarcoma, especially when mainly round cells are present [6], metastatic small cell carcinoma when older patients are affected with an appropriate clinical constellation, and plasmacytoma or primary non-Hodgkin lymphoma of bone in older patients [7].

**Table 3:** Differential diagnoses of small cell osteosarcoma [2]

Entity	Epidemiology	Location	Radiology	Morphology	IHC	Genetics
SCOS	F ≥ M; 2nd decade, > 40	Femur > tibia > humerus > jaws; metaphysis, (diaphysis)	Lytic permeative bone destruction; periosteal reaction; extra-osseous soft tissue mass	Small round-oval-spindle cells; inconspicuous nucleoli; lace-like osteoid (can be focal)	**Positive (variable)**: SATB2, CD99, S100, Actins **Negative**: Cytokeratin, MUC4, EMA (rarely), NKX3.1 (rarely)	Complex chromosomal changes; chromoanagenesis; inter- and intratumoral heterogeneity
Ewing sarcoma	M ≥ F; 2nd decade	Bone (~ 88%): diaphysis and diaphyseal-metaphyseal portion of long bones, pelvis, ribs	Poorly defined osteolytic permeative lesion with onion-skinning or sunburst reaction of periost	Uniform small round cells; inconspicuous nucleoli	**Positive:** CD99 (strong homogeneous membranous), NKX2.2 **Variable:** Cytokeratin, neuro-endocrine markers, S100	*FET::ETS* (most commonly *EWSR1::FLI1*)
Sarcoma with *BCOR* alteration	M > > F; < 20 years	Bone ≥ soft tissue; pelvis, lower extremity, paraspinal	Poorly defined osteolytic or osteosclerotic permeative lesion with/without soft tissue extension	Uniform small round-ovoid-spindled cells; inconspicuous nucleoli; solid sheets/vague nests/short fascicles; rich capillary network; myxoid matrix; osteoid deposition	**Positive:** BCOR, SATB2, TLE1, CyclinD1, and CD99 (variable)	*BCOR* rearrangement (various fusion partners)
Sarcoma with *CIC* rearrangement	M ≥ F; 3rd/4th decade	Soft tissue > visceral > bone (< 5%)	Poorly defined, osteolytic lesion with soft tissue extension [37]	Undifferentiated round cells; vesicular chromatin and prominent nucleoli; diffuse sheets with lobulated growth pattern; necrosis common; brisk mitotic activity	**Positive:** CD99 (patchy), WT1, ETV4 **Variable:** Calretinin, ERG **Negative:** NKX2.2	*CIC-DUX4* (> 95%); rarely non-DUX4 partner genes
Sarcoma with *EWSR1/FUS::NFATC2* rearrangement	M > > F; 3rd decade	Bone (~ 80%): femur > humerus > radius > tibia; metaphysis, diaphysis	Poorly defined osteolytic permeative lesion	Small-medium round-spindled cells; cords/nests/trabeculae/pseudoacinar structures; fibro/myxohyaline stroma	**Positive:** Cytokeratin (dot-like), NKX3.1 **Variable:** CD99, NKX2.2, PAX7, CD138 (rarely)	*EWSR1/FUS::NFATC2; EWSR1* amplification
Mesenchymal chondrosarcoma	M ≥ F; 2nd/3rd decade	Bone (~ 60%): craniofacial, ribs/chest wall, ilium, vertebrae, lower extremity (mostly femur)	Poorly defined osteolytic permeative lesion; mottled calcifications	Small-medium round-spindled cells; hemangiopericytoma-like vasculature; island of well-differentiated hyaline cartilage; matrix may mimic osteoid	**Positive:** CD99 (variable), S100, SOX9, NKX3.1 **Variable:** Desmin, MyoD1, Myogenin, EMA	*HEY1::NCOA2* ( most frequent); alternatively *IRF2BP2::CDX1*
Sclerosing epithelioid fibrosarcoma	M = F; middle-aged to elderly	Soft tissue > > bone	Lytic, expansile lesion with sclerotic rim	Monotonous epithelioid cells; cords/nests/sheets; prominent sclerotic, hyalinized stroma; calcification and chondro-osseous differentiation (sometimes)	**Positive:** MUC4, EMA **Variable:** SMA	*EWSR1::CREB3L1* (most frequent); alternatively *FUS/PAX5::CREB3L1/CREB3L2/CREB3L3/CREM*
Synovial sarcoma	M = F; 2nd/3rd decade	Soft tissue > > bone	Lytic, expansile lesion; can have cortical destruction with extra-osseous extension [38]	Monomorphic primitive spindled cells; monophasic or biphasic	**Positive:** SSX, SS18-SSX, EMA, Cytokeratin (focal), BCL2, CD56, CD99 (weak), S100 (focal), TLE1	*SS18::SSX(1,2,4)*
Lymphoma	Wide age range	Primary bone (< 1% of all non-Hodgkin lymphomas): metadiaphyseal; femur > pelvis > vertebrae > humerus; multifocal	Lytic, permeative lesion with onion-skinning of periost	Small round – larger blastoid cells	Variable; CD45 often positive	Variable
Plasmacytoma of bone	M > F; median age 55 years	Vertebrae > rib > skull > pelvis > femur > humerus > clavicula > scapula	Lytic bone lesion (~ 70%); osteoporosis; pathological fracture	Clusters-sheets of plasma cells	**Positive:** similar to plasma cell myeloma: CD138, CD56, CD117, cyclin D1, monotypic kappa/lambda expression **Negative:** B-cell markers (usually)	Diverse

Abbreviations: *SCOS*, small cell osteosarcoma; *IHC*, immunohistochemistry; *NA*, not applicable

By methylation profiling, 8/18 tumors (44%) were classified as COS, emphasizing that SCOS is a morphological variant of COS as documented in the current WHO classification [2]. It seems that methylation profiling is a robust and reliable test to diagnose (S)COS and to rule out morphological mimics, although fusion gene analysis or the use of immunohistochemical surrogate markers is generally recommended to confirm the diagnosis of fusion-driven neoplasms [16, 35]. Then, 10/18 (56%) cases clustered with the mentioned FDRCS and fusion gene analysis was matching, except in one Ewing sarcoma where no fusion gene was found by anchored multiplex PCR-based NGS, which might be due to an alternative gene rearrangement, e.g. as recently reported [36]. In two cases, one SEF and one mesenchymal chondrosarcoma, RNA quality was insufficient for fusion gene analysis, although in the SEF, *EWSR1* rearrangement using FISH and MUC4 positivity confirmed the diagnosis.

In conclusion, our data, based on DNA-methylation profiling, underpin that SCOS is a morphological variant of COS, sharing overlapping clinical, radiological, morphological, and immunohistochemical features with FDRCS. As biology and consequently treatment modalities differ, molecular analysis is an important diagnostic tool for accurate diagnosis and consequently proper therapeutic strategies, with methylation profiling being a robust and reliable approach when only decalcified FFPE material is available.
